# The Effect of Toric Intraocular Lens Implantation in Irregular Corneal Steep and Flat Meridian

**DOI:** 10.1155/2021/3630668

**Published:** 2021-11-05

**Authors:** Ho Sik Hwang, Hyun Seung Kim, Man Soo Kim, Eun Chul Kim

**Affiliations:** Department of Ophthalmology, College of Medicine, Catholic University of Korea, Seoul, Republic of Korea

## Abstract

**Purpose:**

To evaluate the effect of toric intraocular lens implantation in cataract patients with irregular corneal steep and flat meridian.

**Methods:**

Data of 112 eyes of 78 patients who underwent toric intraocular lens implantation were analyzed retrospectively. Steep meridian deviations (not 180°) and steep and flat meridian deviations (not 90°) were classified as 0, 1–9, 10–19, 20–29, 30–39, and over 30°. Meridian deviation was measured with a sagittal map of a rotating Scheimpflug camera (Pentacam®: Oculus, Wetzlar, Germany) using PicPickTools (NGWIN, Seoul, Korea).

**Results:**

Residual astigmatism (*D*) of 0 (0.51 ± 0.13, 0.55 ± 0.15) and 1–9 (0.61 ± 0.16, 0.66 ± 0.19) groups were significantly lower than that of 10–19 (0.92 ± 0.24, 0.90 ± 0.28), 20–29 (0.10 ± 0.32, 1.01 ± 0.35), and over 30° groups (1.12 ± 0.37, 1.14 ± 0.40) both in steep meridian deviations and horizontal and vertical meridian deviations at 6 months (*P* < 0.05). Postoperative mean UCVA (logMAR) of 0 (0.09 ± 0.04, 0.09 ± 0.05) (logMAR) and 1–9 (0.10 ± 0.04, 0.11 ± 0.08) groups was significantly improved compared to that of 10–19 (0.14 ± 0.05, 0.17 ± 0.10), 20–29 (0.18 ± 0.08, 0.21 ± 0.10), and over 30° groups (0.20 ± 0.09, 0.22 ± 0.11) both in steep meridian deviations and horizontal and vertical meridian deviations at 6 months (*P* < 0.05).

**Conclusions:**

Correction of astigmatism with toric intraocular lens implantation is not accurate in corneas with steep meridian deviations and steep and flat meridian deviations of more than 10°. Therefore, care should be taken when we perform toric intraocular lens implantation in patients with irregular corneal meridian.

## 1. Introduction

Cataract is the most common cause of visual impairment in elderly people [1]. About 60% of patients who have undergone cataract surgery have over 0.75 diopters (*D*) of corneal astigmatism [2], and 20% of patients have astigmatism 1.5 *D* or greater [3]. If this astigmatism is not uncorrected, it results in reduced visual acuity and increased spectacle dependence even after cataract surgery [4]. Toric intraocular lens (toric IOL) is made for not only replacing opaque lens but also correcting corneal astigmatism [1]. Toric IOL is designed for correcting regular corneal astigmatism such that the angle of steep and flat meridian in the cornea is exact 90°. However, not all corneas have regular astigmatism. There may be a deviation of astigmatism meridian even in normal corneas. In this case, steep meridian is not a linear shape (180°) or angle of steep and flat meridian is not 90°. One degree of misalignment of toric IOL causes a loss of approximately 3% of effective cylinder power. The entire toric effect is lost in cases with 30° of misalignment of toric IOL [5]. Therefore, if there is 45° of corneal steep meridian deviations or steep and flat meridian deviation, the toric IOL effect may be lost in spite of perfect alignment of toric IOL without toric IOL rotation postoperatively [6]. Toric IOL implantation can improve visual acuity properly in patients with regular corneal astigmatism.

To the best of our knowledge, no studies have reported the efficacy of toric IOL implantation in normal patients with irregular corneal astigmatism such as steep meridian deviations or steep and flat meridian deviation. Thus, the objective of this study was to evaluate outcomes of toric IOL implantation according to the amount of steep meridian deviations and angle deviation of steep and flat meridian.

## 2. Methods

We performed a retrospective chart review and data analysis in this study. This study was conducted in compliance with Institutional Review Board regulations, informed consent regulations, sponsor and investigator obligations, and the Declaration of Helsinki. The Institutional Review Board (IRB)/Ethics Committee of Bucheon St. Mary Hospital approved this study protocol.

## 3. Patients

A total of 112 eyes of 78 patients who underwent toric intraocular lens implantation in Bucheon St. Mary Hospital from July 2017 to April 2018 were enrolled. Steep meridian deviations (not 180°) were classified as 0, 1–9, 10–19, 20–29, 30–39, and over 30°. Steep and flat meridian deviations (not 90°) were classified as 0, 1–9, 10–19, 20–29, and over 30°. Inclusion criteria were advanced cataracts in patients with high corneal astigmatism more than 1.5D. Exclusion criteria were a history of any ocular injury or disorder, infection, inflammation, and surgery within prior 6 months.

## 4. Axis Evaluation

**Figure 1:**
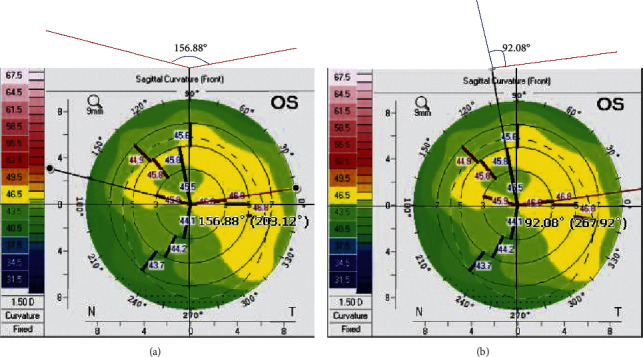
Steep and flat meridian deviation (a) and steep and flat meridian deviation (b). The steep meridian deviation was 23.12° l180–156.88 l, and the steep and flat meridian deviation was 2.08° l90–92.08 l.

Steep meridian deviations and steep and flat meridian deviation were measured with a sagittal map of a rotating Scheimpflug camera (Pentacam®: Oculus, Wetzlar, Germany) using PicPickTools (NGWIN, Seoul, Korea). Steep meridian deviation was determined as an absolute value of 180 minus the steep axis angle. Steep and flat meridian deviation was determined as an absolute value of 90 minus steep and flat meridian angle (Figure 1).

## 5. Preoperative Evaluation

All patients underwent a complete ophthalmological examination. Their demographic and perioperative data were recorded. Uncorrected and corrected distance visual acuities were expressed as logMAR. Manifest refraction, biometry, and keratometry with the IOLMaster partial coherence interferometry device (Carl Zeiss Meditec AG), corneal topography (Pentacam®, Oculus, Germany), slit lamp examination, and dilated funduscopy were examined at the preoperative period and postoperative 2, 4, and 6 months. The IOL manufacturer's web-based toric calculator was used to determine the required cylinder power and axis for the IOL that was going to be implanted. The total corneal astigmatism was measured using the Scheimpflug system (Pentacam^®^, Oculus, Germany).

## 6. Operative Procedures

Before surgery, the corneal limbus was marked at 0°, 90°, and 180° meridian with the patient in a sitting position after instilling topical anesthetic eye drops. All operations were performed under topical anesthesia by a single skilled surgeon (E. C. K) using an Intrepid Infiniti system (Alcon Laboratories, Inc., Fort Worth, TX, USA). Corneal steep axis and 6.0 mm ring were marked with gentian violet. Surgery was performed through a clear corneal incision at the steep astigmatic axis. After topical ocular anesthesia was applied, a 2.75 mm clear corneal incision was made using a 2.75 mm double-blade keratome (Alcon). Surgically induced astigmatism was set by 0.5 diopters. Sodium hyaluronate 1.0% (Hyal Plus, LG Life Science, Seoul, Korea) was used to reform and stabilize the anterior chamber. A continuous curvilinear capsulotomy was made according to a 6.0 mm corneal marker using Inamura capsulorhexis forceps (Duckworth & Kent Ltd., Baldock, UK). Hydrodissection and hydrodelineation were achieved using a balanced salt solution. Phacoemulsification was performed using 2.75 mm-sized phacotips and infusion/aspiration (I/A) cannulas for micro- and small-incision groups, respectively. A clear preloaded IOL (Tecnis ZCT; Abbott Medical Optics) was implanted in the capsular bag. The IOL was rotated to the correct axis position according to the axis of total corneal astigmatism. The wound was not sutured. Postoperative treatment consisted of gatifloxacin 0.3% (Gatiflo, Handok, Chungbuk, Korea) and fluorometholone acetate 0.01% (Ocumetholone, Samil, Seoul, Korea) eye drops four times a day for four weeks.

## 7. Statistical Analysis

All statistical analyses were performed using a commercial program (SPSS for Windows; version 21.0.1; SPSS Inc., Chicago, IL). The Wilcoxon signed rank test was used to compare pre- and postoperative BCVA and refractive and keratometer astigmatisms. *P* values < 0.05 were considered statistically significant.

## 8. Results

### 8.1. Study Population

A total of 112 eyes of 78 patients were enrolled in this study. Preoperative mean autorefractive cylinder was 2.21 ± 1.36 D. Mean total corneal astigmatism measured with the Scheimpflug camera was 2.35 ± 1.15 D. Mean UCVA was 0.83 ± 0.35 (logMAR), and mean BCVA was 0.56 ± 0.29 (logMAR) (Table 1).

Preoperative mean estimated astigmatism of all patients was 0.02 ± 0.12 D. Mean autorefractive cylinder at two months after toric IOL implantation (0.57 ± 0.31 D) was significantly decreased compared to its preoperative value (2.21 ± 1.36 D) (*P* < 0.05). Postoperative mean total corneal astigmatism (1.96 ± 1.0 D) was also decreased compared to preoperative value (2.35 ± 1.15 D), but the decrease was statistically insignificant. Mean IOL rotation was 3.2 ± 1.5° at 2 months after toric IOL implantation. Postoperative mean UCVA (0.09 ± 0.06) (logMAR) and mean BCVA (0.02 ± 0.01) were improved compared to preoperative values (0.83 ± 0.35, 0.56 ± 0.29, respectively) (both *P* < 0.05) (Table 2).

### 8.2. Steep Meridian Deviation

**Figure 2:**
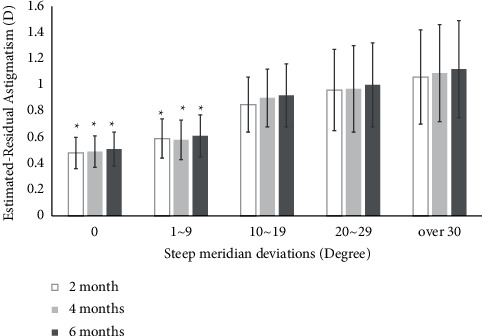
Postoperative estimated residual astigmatism according to steep meridian deviation. Values are presented as mean ± SD. Estimated residual astigmatism D values of 0 (0.48 ± 0.12) and 1–9° groups (0.59 ± 0.15) were significantly lower than those of 10–19 (0.85 ± 0.21), 20–29 (0.96 ± 0.31), and over 30° groups (1.06 ± 0.36) in steep meridian deviations at postoperative 2, 4, and 6 months (*P* < 0.05).

**Figure 3:**
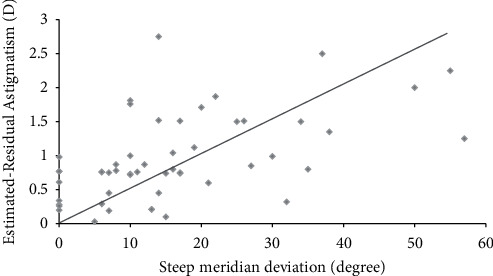
Pearson's correlation between steep meridian deviation and estimated residual astigmatism. There was a positive correlation between steep meridian deviation and the estimated residual astigmatism D at 6 months (*r* = 0.47, *P* < 0.05).

In steep meridian deviation, preoperative total corneal astigmatism (*D*) and mean UCVA (logMAR) of all groups were similar to each other (all *P* > 0.05). Postoperative mean UCVA (logMAR) of 0 (0.09 ± 0.04) (logMAR) and 1–9° (0.10 ± 0.04) groups was significantly improved compared to that of 10–19 (0.14 ± 0.05), 20–29 (0.18 ± 0.08), and over 30° groups (0.20 ± 0.09) in steep meridian deviations at 6 months (all *P* < 0.05) (Table 3). Estimated residual astigmatism (*D*) values of 0 (0.51 ± 0.13) and 1–9° groups (0.61 ± 0.16) were significantly lower than those of 10–19 (0.92 ± 0.24), 20–29 (0.10 ± 0.32), and over 30° groups (1.12 ± 0.37) in steep meridian deviations at postoperative 6 months (*P* < 0.05) (Figure 2). There was a positive correlation between steep meridian deviation and the estimated residual astigmatism (*D*) (*r* = 0.47, *P* < 0.05) (Figure 3).

### 8.3. Steep and Flat Meridian Deviation

**Figure 4:**
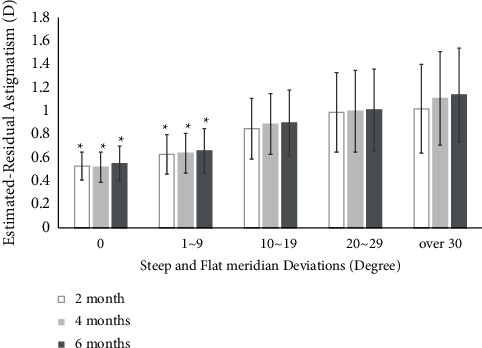
Postoperative estimated residual astigmatism according to steep and flat meridian deviations. Values are presented as mean ± SD. D, diopter. Estimated-residual astigmatism D values of 0 (0.53 ± 0.12) and 1–9° groups (0.63 ± 0.17) were significantly lower than those of 10–19 (0.85 ± 0.26), 20–29 (0.99 ± 0.34), and over 40° groups (1.02 ± 0.38) in steep and flat meridian deviations at postoperative 2, 4, and 6 months (*P* < 0.05).

**Figure 5:**
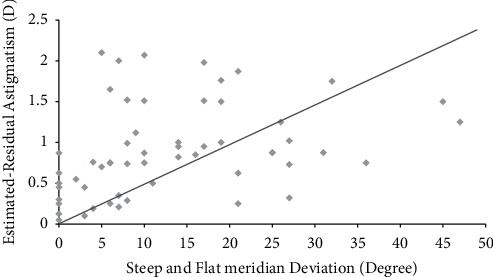
Pearson's correlation between steep and flat meridian deviation and estimated residual astigmatism. There was a positive correlation between steep and flat meridian deviation and the estimated residual astigmatism D at 6 months (*r* = 0.45, *P* < 0.05).

In steep and flat meridian deviation, preoperative mean UCVA (logMAR) values of all groups were similar to each other (*P* > 0.05). However, preoperative total corneal astigmatism (*D*) of 21–30° (2.04 ± 0.86 D) and over 30° groups (2.06 ± 1.05 D) was statistically higher than that of other groups (1.47 ± 0.72, 1.68 ± 0.24, and 1.71 ± 0.40, respectively) (*P* < 0.05). Postoperative mean UCVA (logMAR) values of 0 (0.09 ± 0.05) and 1–9° (0.11 ± 0.08) were significantly improved compared to those of 10–19 (0.17 ± 0.10), 20–29 (0.21 ± 0.10), and over 30° groups (0.22 ± 0.11) in steep and flat meridian deviations at 6 months (*P* < 0.05) (Table 4). Estimated residual astigmatism (*D*) of 0 (0.55 ± 0.15) and 1–9° groups (0.66 ± 0.19) was significantly lower than that of 10–19 (0.90 ± 0.28), 20–29 (1.01 ± 0.35), and over 30° groups (1.14 ± 0.40) in steep and flat meridian deviations at postoperative 6 months (*P* < 0.05) (Figure 4). There was a positive correlation between steep and flat meridian deviations and estimated residual astigmatism (*D*) (*r* = 0.45, *P* < 0.05) (Figure 5).

## 9. Discussion

Implantation of toric IOL in cataract surgery to correct corneal astigmatism has been popular due to its excellent clinical outcomes and increased patient demands [7]. However, the uncorrected visual acuity of patients with toric IOLs implantation is variable according to postoperative residual astigmatism. The range of residual astigmatism after toric IOL implantation has been reported to be 0.00 to 2.25 *D* depending on preoperative astigmatism [8]. Several factors can increase residual astigmatism after toric IOL implantation, including misalignment of toric IOL, rotation of toricIOL [7], ignoring posterior corneal astigmatism [9], inaccurate measurements of the cornea, incorrect power of the toric IOL, and inaccurate surgically induced astigmatism. Postoperative rotation of the toric IOL is the most important factor associated with increasing residual astigmatism [10]. Zhu et al. have reported that the mean absolute rotation of the IOL is 8.83 ± 5.26° [10]. In the present study, average toric IOL rotation was 3.2 ± 1.5° (Table 2).

If there are severe steep meridian deviations, the other marker of toric IOL and corneal steep meridian may be misaligned despite that surgeons have made a perfect alignment of one marker of toric IOL and corneal steep meridian. If there is a severe steep and flat meridian deviation, the toric IOL steep axis and corneal flat axis may be misaligned despite that the surgeon has made a perfect alignment of marker of toric IOL and corneal steep meridian.

In this study, estimated residual astigmatism (*D*) values of 0 (0.51 ± 0.13) and 1–9° groups (0.61 ± 0.16) were significantly lower than those of 10–19 (0.92 ± 0.24), 20–29 (0.10 ± 0.32), and over 30° groups (1.12 ± 0.37) in steep meridian deviations at postoperative 6 months (*P* < 0.05) (Figure 2). Preoperative total corneal astigmatism (*D*) and mean UCVA (logMAR) values of all groups were similar to each other (*P* > 0.05). However, postoperative mean UCVA (logMAR) of 0 (0.09 ± 0.04) (logMAR) and 1–9° (0.10 ± 0.04) groups was significantly improved compared to that of 10–19 (0.14 ± 0.05), 20–29 (0.18 ± 0.08), and over 30° groups (0.20 ± 0.09) in steep meridian deviation at 6 months (*P* < 0.05) (Table 3).

In steep and flat meridian deviation, preoperative total corneal astigmatism (*D*) values of 21–30° and over 30° groups were statistically higher than those of other groups (*P* < 0.05) (Table 3). We hypothesize that steep and flat meridian deviation would make more irregular astigmatism than steep meridian deviation if the deviation angle is more than 21°.

Estimated residual astigmatism (*D*) of 0 (0.55 ± 0.15) and 1–9° groups (0.66 ± 0.19) were significantly lower than that of 10–19 (0.90 ± 0.28), 20–29 (1.01 ± 0.35), and over 30° groups (1.14 ± 0.40) in steep and flat meridian deviations at postoperative 6 months (*P* < 0.05) (Figure 4). Also, postoperative mean UCVA (logMAR) values of 0 (0.09 ± 0.05) and 1–9° (0.11 ± 0.08) were significantly improved compared to those of 10–19 (0.17 ± 0.10), 20–29 (0.21 ± 0.10), and over 30° groups (0.22 ± 0.11) in steep and flat meridian deviations at 6 months (*P* < 0.05) (Table 4).

There was a positive correlation between steep meridian deviation and the estimated residual astigmatism (*D*) at 6 months (*r* = 0.47, *P* < 0.05) (Figure 3). A positive correlation between steep and flat meridian deviation and estimated residual astigmatism (*D*) (*r* = 0.45, *P* < 0.05) (Figure 5) was also found at 6 months.

Toric IOL rotations of less than 10° changed the eye's refractive astigmatism to less than 0.50 diopters [11]. In the present study, postoperative mean UCVA (logMAR) values of 0° and 1–9° groups were significantly improved compared to those of other groups both in steep meridian deviation (Table 3) and in steep and flat meridian deviation (Table 4) (*P* < 0.05). Therefore, patients who had meridian deviations of less than 10° had significantly lower residual astigmatism and better uncorrected visual acuity than those who had meridian deviation of more than 10°.

To the best of our knowledge, this was the first study to evaluate the outcomes of toric IOL implantation in cataract patients with irregular corneal astigmatism. The postoperative visual acuity of patients with regular astigmatism was significantly improved compared to that of patients with steep meridian deviations and the steep and flat meridian deviations. If steep meridian deviations and steep and flat meridian deviations increased, estimated residual astigmatism (*D*) also increased in patients with irregular corneal astigmatism. Therefore, the effect of toric IOL implantation is optimized in patients with regular corneal astigmatism. Toric IOL implantation should be performed cautiously when patients have steep meridian deviations or steep and flat meridian deviations.

## 10. Study Limitations

A multicenter clinical trial with a larger sample size and longer follow-up period is needed to observe the long-term efficacy of toric intraocular lens implantation in cataract patients with irregular corneal astigmatism.

## Figures and Tables

**Table 1:** The patient demographics and the preoperative data.

Parameters	
Total patients (eyes)	112 (78)
Male:women (ratio)	74 : 38
Patient age (years)	67.7 ± 12.8
Mean autorefractive cylinder (D)	2.21 ± 1.36
Mean total corneal astigmatism (D)	2.35 ± 1.15
Mean UCVA (logMAR)	0.83 ± 0.35
Mean BCVA (logMAR)	0.56 ± 0.29

Data represent mean ± standard deviation. D : diopter. General results after two months are shown.

**Table 2:** Postoperative results of patient 2 months after toric IOL implantation.

Parameters	
Preoperative mean estimated astigmatism (D)	0.02 ± 0.12
Mean autorefractive cylinder (D)	0.57 ± 0.31
Mean total corneal astigmatism (D)	1.96 ± 1.0
Mean IOL rotation (degree)	3.2 ± 1.5
Mean UCVA (logMAR)	0.09 ± 0.06
Mean BCVA (logMAR)	0.02 ± 0.01

Data represent mean ± standard deviation. D : diopter

**Table 3:** Preoperative and postoperative total corneal astigmatism (*D*) and uncorrected visual acuity (UCVA) according to the steep meridian deviation.

Parameters	0 degree	1–9 degrees	10–19 degrees	20–29 degrees	Over 30 degrees
Eyes (n)	20	30	32	18	12
Preop total corneal astigmatism (D)	1.72 ± 0.90	1.85 ± 0.90	1.96 ± 0.64	2.08 ± 1.39	2.13 ± 1.05
Preop mean UCVA (logMAR)	0.40 ± 0.15	0.42 ± 0.13	0.41 ± 0.14	0.42 ± 0.21	0.43 ± 0.17
2-month postop mean UCVA (logMAR)	0.08 ± 0.03^*∗*^	0.09 ± 0.04^*∗*^	0.13 ± 0.04	0.16 ± 0.05	0.19 ± 0.07
4-month postop mean UCVA (logMAR)	0.09 ± 0.03^*∗*^	0.09 ± 0.03^*∗*^	0.15 ± 0.06	0.15 ± 0.09	0.21 ± 0.11
6-month postop mean UCVA (logMAR)	0.09 ± 0.04^*∗*^	0.10 ± 0.04^*∗*^	0.14 ± 0.05	0.18 ± 0.08	0.20 ± 0.09
6-month Postop mean UCVA (logMAR)	0.09 ± 0.04^*∗*^	0.10 ± 0.04^*∗*^	0.14 ± 0.05	0.18 ± 0.08	0.20 ± 0.09

Data represent mean ± standard deviation. D; diopter, ^*∗*^*P* < 0.05

**Table 4:** Preoperative and postoperative total corneal astigmatism (*D*) and uncorrected visual acuity (UCVA) according to the steep and flat meridian deviation.

Parameters	0 degree	1–9 degrees	10–19 degrees	20–29 degrees	Over 30 degrees
Eyes (n)	16	46	22	18	10
Preop total corneal astigmatism (D)	1.47 ± 0.72	1.68 ± 0.24	1.71 ± 0.40	2.04 ± 0.86^*∗*^	2.06 ± 1.05^*∗*^
Preop mean UCVA (logMAR)	0.35 ± 0.13	0.40 ± 0.14	0.42 ± 0.17	0.43 ± 0.20	0.45 ± 0.20
2-month postop mean UCVA (logMAR)	0.08 ± 0.03^*∗*^	0.08 ± 0.04^*∗*^	0.15 ± 0.05	0.17 ± 0.05	0.18 ± 0.06
4-month postop mean UCVA (logMAR)	0.10 ± 0.07^*∗*^	0.09 ± 0.05^*∗*^	0.17 ± 0.09	0.18 ± 0.11	0.20 ± 0.09
6-month postop mean UCVA (logMAR)	0.09 ± 0.05^*∗*^	0.11 ± 0.08^*∗*^	0.17 ± 0.10	0.21 ± 0.10	0.22 ± 0.11

Data represent mean ± standard deviation. D; diopter, ^*∗*^*P* < 0.05

## Data Availability

The data used to support the findings of this study are available from the corresponding author upon request.
